# ‘*I still can’t forget those words*’: mixed methods study of the persisting impact on patients reporting psychosomatic and psychiatric misdiagnoses 

**DOI:** 10.1093/rheumatology/keaf115

**Published:** 2025-03-03

**Authors:** Melanie Sloan, Michael Bosley, Caroline Gordon, Thomas A Pollak, Farhana Mann, Efthalia Massou, Stephen Morris, Lynn Holloway, Rupert Harwood, Kate Middleton, Wendy Diment, James Brimicombe, Elliott Lever, Lucy Calderwood, Ellie Dalby, Elaine Dunbar, David D’Cruz, Felix Naughton

**Affiliations:** Primary Care Unit, Department of Public Health and Primary Care, University of Cambridge, Cambridge, UK; Patient and Public Partners; Rheumatology Research Group, Institute of Inflammation and Ageing, College of Medical and Dental Sciences, University of Birmingham, Birmingham, UK; Institute of Psychiatry, Psychology and Neuroscience, King’s College London, and SLAM NHS Foundation Trust, London, UK; Department of Psychiatry, UCLH, London, UK; Primary Care Unit, Department of Public Health and Primary Care, University of Cambridge, Cambridge, UK; Primary Care Unit, Department of Public Health and Primary Care, University of Cambridge, Cambridge, UK; Patient and Public Partners; Swansea University Medical School, Wales, UK; Patient and Public Partners; Patient and Public Partners; Primary Care Unit, Department of Public Health and Primary Care, University of Cambridge, Cambridge, UK; Rheumatology Department, Whittington Hospital, London, UK; Patient and Public Partners; Patient and Public Partners; Patient and Public Partners; The Louise Coote Lupus Unit, Guy’s and St Thomas’ Hospitals NHS Foundation Trust, London, UK; Lifespan Health Research Centre, University of East Anglia, Norwich, UK

**Keywords:** misdiagnoses, psychosomatic, psychiatric, mental health, healthcare-seeking behaviours, healthcare avoidance, patient–clinician relationships

## Abstract

**Objectives:**

This research aimed to improve understanding of persisting impacts of patient-reported psychosomatic and psychiatric misdiagnoses on patients with systemic autoimmune rheumatic diseases (SARDs).

**Methods:**

Mixed methods data from two SARDs cohorts were analysed (*N* = 1543 and *N* = 1853). Validated instruments and patient-designed questions were used to measure self-reported depression, anxiety and mental wellbeing, in addition to medical relationships and healthcare behaviours. Comparative tests were used to evaluate differences between patients reporting psychosomatic and/or psychiatric misdiagnoses and other patients.

**Results:**

Persisting adverse outcomes of perceived psychosomatic and psychiatric misdiagnoses were identified in multiple domains. This included >80% of patients reporting that it had damaged their self-worth, and 72% reporting that it still upset them. Patients reporting psychosomatic and/or psychiatric misdiagnoses had significantly lower mental wellbeing, and higher depression and anxiety levels (all *P* < 0.001), and lower levels of satisfaction with every aspect of medical care, compared with patients reporting no psychosomatic or psychiatric misdiagnoses. Psychosomatic and psychiatric misdiagnoses had varying associations with healthcare behaviours, including a significantly higher likelihood of under-reporting symptoms (*P* < 0.001) and healthcare avoidance (*P* = 0.012), but not with medication adherence (*P* = 0.2). Thematic analysis of qualitative data revealed that symptom under-reporting and healthcare avoidance often resulted from distrust and fear that symptoms would be disbelieved and misattributed again.

**Conclusion:**

Patient-reported psychosomatic and psychiatric (mis)diagnoses are associated with persisting adverse impacts in multiple domains including mental health, medical relationships, self-worth, and some healthcare behaviours. Health services and clinicians should consider these potential adverse impacts on patients and offer support to reduce any persisting negative impacts.

Rheumatology key messages

- Psychosomatic and psychiatric misdiagnoses are associated with persisting adverse impacts in multiple domains, including patient wellbeing.
- The persisting impacts of psychosomatic and psychiatric misdiagnoses need recognition, clinician–patient discussion, and support.
- Psychosomatic and psychiatric misdiagnoses may be an iatrogenic *cause* of mental health symptoms.

## Introduction

The journey from symptom onset to diagnosis of a systemic autoimmune rheumatic disease (SARD) can be long [1–4] and challenging for patients and clinicians. Previous studies identified that around 50% of systemic lupus erythematosus (SLE) patients were initially misdiagnosed [1], with patients often reporting accruing—sometimes multiple—psychosomatic and/or psychiatric (mis)diagnoses before and after diagnosis [5]. Adverse impacts of diagnostic delays and misdiagnoses have been recognized in SARDs [6–11], including explicit [10] and implicit [11] references to iatrogenic harm. These impacts have been found to be particularly apparent in patients feeling that their symptoms were treated dismissively by physicians, including as medically unexplained symptoms (MUS) [7]. The negative impact of delayed treatment from diagnostic delays on future health, disease progression and disability has been reported [6, 12]. However, a gap in the literature exists with regard to the persisting impact of psychosomatic and/or psychiatric misdiagnoses on patient mental health, medical relationships and healthcare behaviours.

While it is widely recognized that the quality of the medical relationship is associated with patient satisfaction [13], and trust and better health outcomes are positively associated [14, 15], understanding of the impact of medical trust remains incomplete, even with well-researched diseases such as cancer [16]. We considered whether any existing theories and models could assist in explaining enduring impacts of psychosomatic and/or psychiatric misdiagnoses on patient and clinician behaviours and interactions. Attachment theory usually focuses on the impact of relationships at an early stage in life on the ability to build secure trusting relationships in the future [17, 18]. There has been limited exploration of how medical relationships at an early stage of SARDs’ diagnostic and disease journeys could have some comparable impacts on building future trusting relationships with clinicians. In addition, the Patient Health Engagement (PHE) model [19] is under-explored in relation to SARDs patients and their medical interactions. This model describes patients moving through four stages (‘blackout’, ‘arousal’, ‘adhesion’ and ‘eudaimonic’) of increasing acceptance and empowerment [20].

We have previously explored the impacts of arduous diagnostic journeys on medical relationships [5, 21] and have also reported on the high prevalence and difficulties in attribution of neuropsychiatric (NP) symptoms in SARDs [22, 23]. Viewed in combination, these findings indicated the importance of further investigations into the extent to which previous misdiagnoses are impacting—or potentially even directly *causing* in some cases—certain mental health symptoms. We used the data from two of our large-scale SARD mixed methods studies (LISTEN [24] and INSPIRE [22]) to quantitatively test the pre-specified hypothesis that psychosomatic and/or psychiatric misdiagnoses are associated with long-term adverse impacts on patients’ future medical relationships, healthcare behaviours and mental wellbeing. The study also aimed to consider the relevance of the PHE model and attachment theory, and to qualitatively further increase understanding of the persisting impact of psychosomatic and/or psychiatric misdiagnoses. This is essential to enable more awareness and support for those experiencing the repercussions of these misdiagnoses, and to reduce the previously reported propensity of some clinicians [5, 21] to assume a psychosomatic or psychiatric cause for the multiple symptoms that SARDs patients may present with.

## Methods

### Participants

Data are from two sources:

1. The LISTEN study (N = 1543 surveys): A cohort of SARD patients completing a survey, and sub-sample completing interviews, predominantly focused on the impact of Covid-19 on care and medical relationships conducted in April 2021 [24].
2. The INSPIRE project (N = 1853 surveys): Surveys and interviews investigating neuropsychiatric symptoms in SARDs conducted in mid-2022 [22].

Both studies recruited participants online through social media, disease support groups, and professional networks. The surveys were hosted via Qualtrics. The studies and their methods have been described in detail previously [22, 25]. Participants were patients aged 18 or over who marked a box on the survey to confirm that they had a SARD diagnosis reported on clinical correspondence. Patient interviewees were purposively selected from both LISTEN and INSPIRE survey responses to ensure that a broad range of diseases, socio-demographic attributes and opinions regarding medical care were represented. Clinicians from a variety of specialities were also surveyed and interviewed for both studies. Only their interview results were used for this study as their surveys did not include questions on misdiagnoses.

### Study design and measures

Both LISTEN and INSPIRE had pre-specified secondary aims to investigate the impact of perceived misdiagnoses on medical relationships, healthcare behaviours and wellbeing. Multi-stage sequential mixed methods were used, as described in the first INSPIRE study [21].

Validated instruments included the Warwick Edinburgh Mental Wellbeing Scale (WEMWBS) [26], a PROMIS measurement for depression (8-item negatively phrased) and the general anxiety disorder index (GAD-7) [27]. Healthcare behaviours investigated included: medication adherence, frequency and accuracy of reporting symptoms, and frequency of self-managing symptoms. Questions investigating perceptions of medical relationships included: trust in clinicians, satisfaction with care, confidence in prompt support and medical security. Response options were generally via 5–7 point Likert-type scales or using a scale from 0–100. For example, medical security was defined to patients as ‘a clinician being there when you need them and able to help you’ with a response scale from 0 to 100.

Our earlier research suggested that the greatest ongoing distress and loss of trust in clinicians was due to perceived psychosomatic and psychiatric misdiagnoses [5, 21, 28], therefore both surveys included questions on misdiagnoses. The LISTEN survey asked if respondents had received any psychosomatic, mental health or ‘in your head’ type misdiagnosis, and a further question asked if they had received a misdiagnosis of an alternative ‘physical’ (referred to henceforth as somatic) disease. The INSPIRE survey listed multiple common SARD diagnoses/misdiagnoses/co-diagnoses which included a combination (randomly ordered) of psychiatric, potentially psychosomatic and somatic conditions. These included: depression, anxiety, psychosis, ME/CFS, functional disorders, stroke, autonomic dysfunction, fibromyalgia and anti-phospholipid syndrome (APS), and whether a clinician had attributed their symptoms to psychosomatic, psychological or ‘lifestyle’ causes. Participants were then asked whether they considered each of their previously listed diagnoses to be correct or to have been a misdiagnosis. Participants who marked ‘not sure’ were excluded. Categorization of misdiagnosis was based on the participant self-reports and their perception of whether or not the clinician’s diagnosis was correct. Any reference to misdiagnoses is based on the patient’s interpretation as the research team had no means to verify these.

Qualitative data were collected via open-ended survey questions (for example, ‘how have relationships (good or bad) with doctors impacted your mental health?’) and through in-depth interviews. Sections included mental health, medical relationships and healthcare behaviours, and the impact of misdiagnoses on these areas. Interviews were carried out mostly by Zoom by experienced medical research interviewers. Interviews were audio-recorded and transcribed verbatim.

## Analysis

### Quantitative analysis

Two groups of participants were compared from each survey in terms of outcomes of interest with analysis following a pre-agreed statistical analysis plan. In the LISTEN cohort, we compared those misdiagnosed with an alternative (to their SARD) somatic disease (*n* = 348) to those with a psychosomatic/psychiatric misdiagnosis (*n* = 347). Of the 1853 INSPIRE survey participants, patients who reported receiving a psychosomatic or psychiatric misdiagnosis (*n* = 378) were compared with those reporting no psychosomatic and/or psychiatric misdiagnosis (maximum of *n* = 1400 depending on survey section response rate). This second group included those participants receiving a psychosomatic/psychiatric diagnosis who believed it to be accurate. Satisfaction with care, healthcare behaviours and mental health measures were assessed in each cohort using descriptive statistics (i.e. mean values and standard deviations).

We investigated whether the difference in mean values between the two groups of each cohort were statistically significant using *t*-tests and Kruskal–Wallis tests as appropriate. An alpha of <0.05 is used as the cut-off for significance.

### Qualitative analysis

Relevant extracts of responses to the open-ended survey questions and interviews were analysed thematically [29], with NVivo12 used to facilitate the coding and organization of the qualitative data. Themes generated directly from the results were discussed and agreed by the wider team, including patient representatives and clinicians. Qualitative results were used to further explain quantitative findings, with particular attention given to divergent findings and cases. A detailed description of our qualitative method is included in the INSPIRE survey supplementary information of the first INSPIRE paper [21].

### Ethical approval

The Cambridge Psychology Research Committee provided ethical approval: PRE.2019.099 and PRE.2020.089 (LISTEN trial and COVID-19-related amendments, ISRCTN-14966097), and PRE 2022.027 (the INSPIRE project, https://osf.io/zrehm).

## Results

Out of the *N* = 1543 LISTEN and *N* = 1853 INSPIRE survey participants (Table 1), the majority were female (>91%). The most frequently reported SARDs were SLE and inflammatory arthritis. Clinician interviewees were from a wide variety of countries with 64% from the UK, and 40% were rheumatologists. Percentages of patients accepting a psychosomatic or psychiatric diagnosis as correct ranged from 7% for psychosomatic symptoms to 86% for depression. Although many patients with all types of misdiagnoses reported long-term physical damage—for example, ‘*lost the sight in one eye*’ (Ppt 300, vasculitis, England)—from the delay and/or mistreatment, this study focused on the persisting effects on patient lives beyond the physical effects.

**Table 1.** Participant characteristics

Characteristic	LISTEN patients’ survey (N = 1543)	INSPIRE patients’ survey (N = 1853)	Patients’ interviews (N = 67)	Clinicians’ interviews (N = 50)
**Age**				
<30	273 (18%)	94 (5%)	6 (9%)	0
30–39	302 (20%)	195 (11%)	5 (7%)	11 (22%)
40–49	460 (30%)	298 (16%)	17 (25%)	19 (38%)
50–59	340 (22%)	519 (28%)	16 (24%)	12 (24%)
60+	168 (11%)	745 (40%)	23 (34%)	8 (16%)
Prefer not to say	0 (0%)	2 (<1%)	0 (0%)	0 (0%)
**Gender**				
Female	1464 (95%)	1687 (91%)	60 (90%)	23 (46%)
Male	71 (4%)	160 (9%)	7 (10%)	27 (54%)
Other/undisclosed	8 (1%)	6 (<1%)	0 (0%)	0 (0%)
**Country/region**				
England	1196 (78%)	1285 (69%)	38 (57%)	28 (56%)
Scotland	139 (9%)	144 (8%)	7 (10%)	2 (4%)
Wales	78 (5%)	104 (6%)	7 (10%)	2 (4%)
N. Ireland or Republic of Ireland	48 (3%)	35 (2%)	3 (4%)	0 (0%)
US or Canada	37 (2%)	112 (6%)	4 (6%)	4 (8%)
Europe	20 (1%)	121 (7%)	4 (6%)	6 (12%)
Other	25 (2%)	52 (3%)	4 (5%)	8 (16%)
**Disease**				
SLE	497 (32%)	566 (31%)	25 (37%)	
Inflammatory arthritis	472 (31%)	456 (25%)	9 (13%)	
Sjögren’s	128 (8%)	150 (8%)	6 (9%)	
Systemic sclerosis	128 (8%)	63 (3%)	2 (3%)	
PMR	57 (4%)	132 (7%)	7 (10%)	
Vasculitis	53 (3%)	200 (11%)	3 (4%)	
UCTD	50 (3%)	77 (4%)	9 (13%)	
MCTD or two or more inflammatory rheumatic diseases	104 (7%)	145 (8%)	3 (4%)	
Other inflammatory rheumatic disease	53 (3%)	64 (4%)	3 (4%)	
**Time since diagnosis**				
<1 year	96 (6%)	183 (7%)	3 (7%)	
1–2 years	187 (12%)	257 (14%)	6 (15%)	
3–5 years	316 (21%)	391 (21%)	7 (17%)	
6–9 years	293 (19%)	340 (18%)	11 (27%)	
10+ years	645 (42%)	714 (39%)	13 (32%)	
Unsure or missing	6 (<1%)	13 (1%)	1 (2%)	
**Clinician role**				
Rheumatologist				20 (40%)
Psychiatrist				8 (16%)
Neurologist				10 (20%)
Rheumatology nurse				4 (8%)
GP/Primary care				5 (10%)
Other speciality				3 (6%)

### Impact on medical relationships

**Figure 1.**
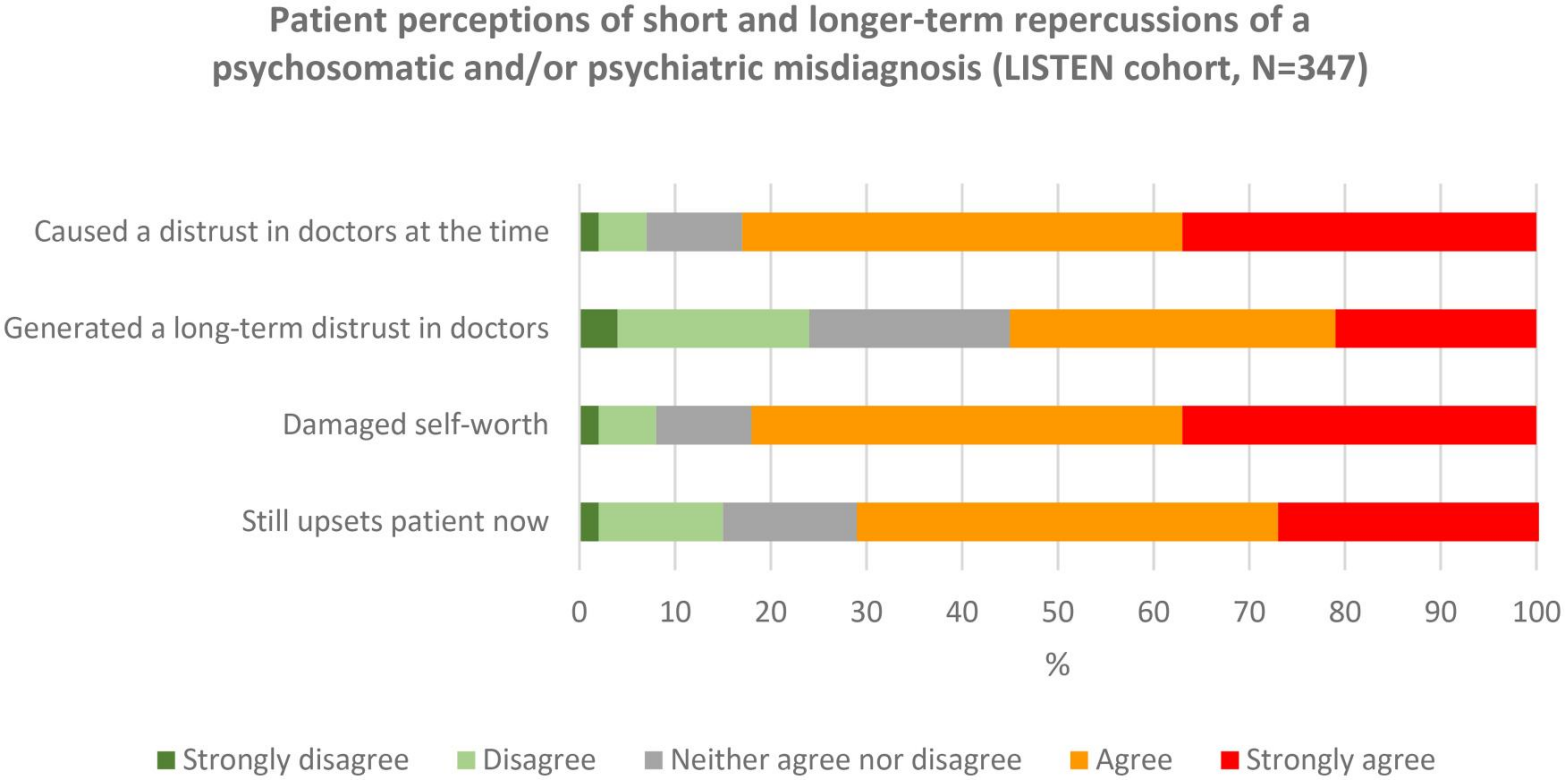
Repercussions of a psychosomatic and/or psychiatric misdiagnosis

Over 80% of patients reporting a psychosomatic and/or psychiatric misdiagnosis reported damaged trust in clinicians at the time of the misdiagnosis, and 55% felt it had engendered a long-term distrust in clinicians (Fig. 1, *n* = 347).

**Figure 2.**
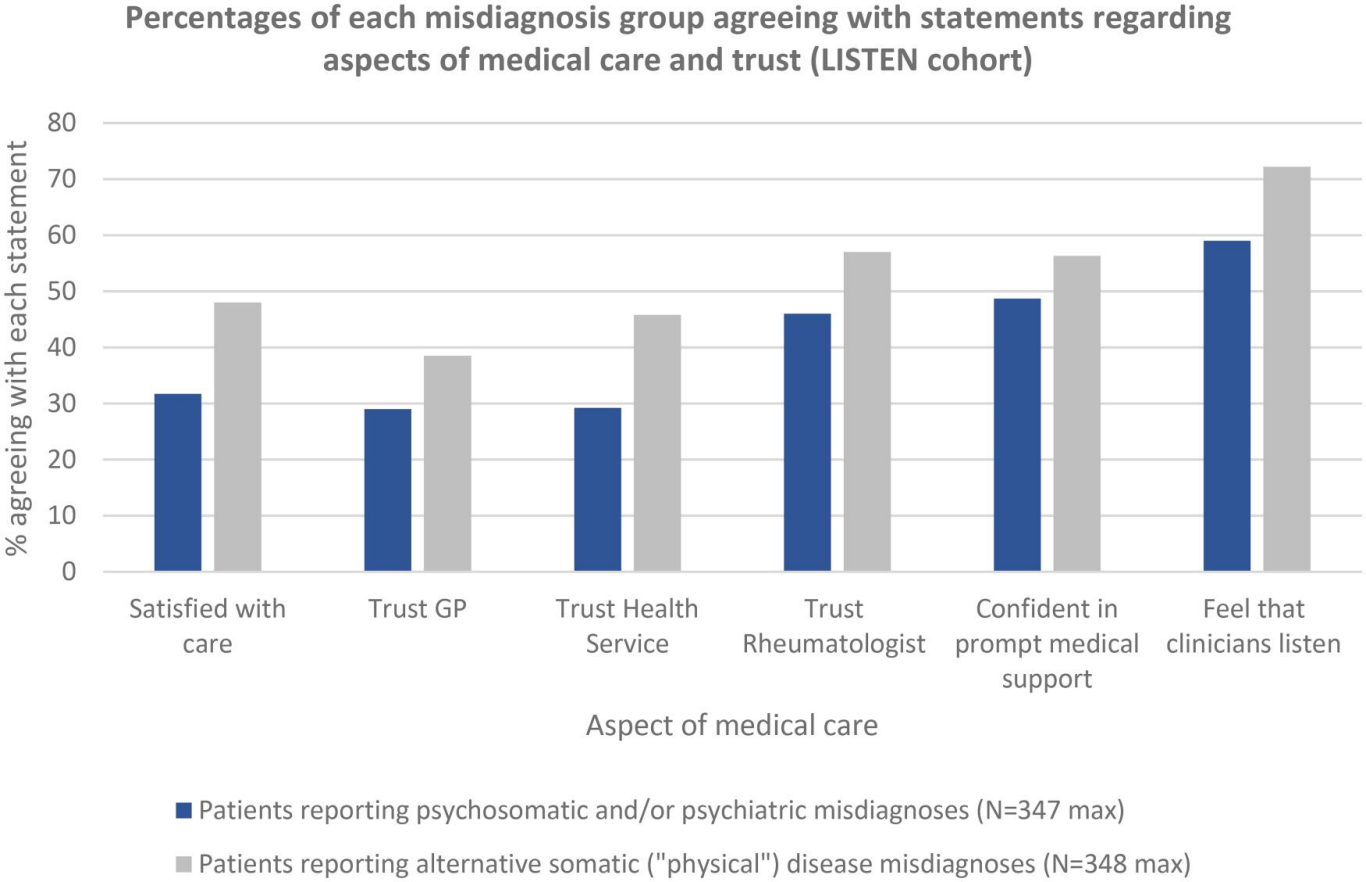
Comparison of medical trust and satisfaction with care by misdiagnosis type. Note: The LISTEN study was during the peak of the Covid-19 pandemic when care was severely disrupted. These percentages are therefore suitable for comparison only rather than as an indication of general satisfaction with care

Significantly lower proportions of patients reporting psychosomatic and/or psychiatric misdiagnoses agreed that they were satisfied with all aspects of care (Fig. 2). These findings were expanded upon by many patients in interviews, with the perception of feeling ‘*disbelieved*’ (multiple patients) indicated to have been particularly impactful on future medical relationships:

‘*It’s so sad it took me developing non-repairable damage to my joints which showed on an MRI before any medical people truly believed me and my pain. I am trying to repair my trust but feel very much gaslighted*’ (Ppt 876, RA, Scotland)

Trust in clinicians in general was lower than trust in the patients’ own clinicians regardless of experience of misdiagnoses. Many patients reported finding a trusting medical relationship at various points after their misdiagnosis, although for some it had taken many attempts: ‘*you have to kiss a lot of frogs to find your prince*’ (Ppt 172, dermatomyositis, England), and many reported greater caution thereafter:

‘*Taught me to calibrate my trust carefully. To date, a specialist I see is my best medical ally … we are making a good team. Do I trust them? Sort of, but not blindly, never blindly*’ (Ppt 731, UCTD, USA)

In the LISTEN cohort (Table 2), the group reporting psychosomatic and/or psychiatric misdiagnoses had significantly lower trust (in GP, rheumatologist and health service, all *P* < 0.001), lower satisfaction with care and perceptions of clinician’s listening skills (both *P* < 0.001), and lower confidence in prompt medical support (*P* = 0.006) than those misdiagnosed with an alternative somatic disease. Similar findings were observed in INSPIRE (Table 3) where significantly lower scores were found among those reporting a previous psychosomatic/psychiatric misdiagnosis regarding satisfaction with medical support, trust in own clinicians and trust in clinicians generally (all *P* < 0.001).

**Table 2.** Differences in perceptions of medical care and relationships between types of misdiagnosis (LISTEN)

Category	Mean (SD) satisfaction score for patients misdiagnosed with an alternative somatic disease (*n* = 348)	Mean (SD) satisfaction score for patients misdiagnosed with a psychosomatic or mental health condition (*n* = 347)	Mean (SE) difference	95% confidence interval	*P* value of *t*-test
Satisfied with care	3.18 (1.14)	2.70 (1.19)	−0.48 (0.09)	−0.65, −0.30	**<0.001**
Confidence in prompt medical support	3.42 (1.05)	3.19 (1.16)	−0.23 (0.08)	−0.40, −0.68	**0.006**
Perception of clinician’s listening	3.88 (0.99)	3.46 (1.18)	−0.42 (0.08)	−0.58, −0.26	**<0.001**
Trust in GP	3.52 (1.54)	3.10 (1.54)	−0.41 (0.12)	−0.64, −0.18	**<0.001**
Trust in rheumatologist	4.30 (1.67)	3.88 (1.75)	−0.42 (0.13)	−0.68, −0.17	**<0.001**
Trust in the health service	3.75 (1.51)	3.22 (1.41)	−0.52 (0.11)	−0.74, −0.30	**<0.001**

Scales were 1–5 (highest level of satisfaction). The significance is written for each value e.g. <0.001 for the first and 0.006 for the second.

**Table 3.** Comparison of satisfaction with care measures between those reporting and those not reporting a psychosomatic or psychiatric misdiagnosis (INSPIRE)

Satisfaction with care measures	Mean (SD) for patients not misdiagnosed with a psychosomatic or psychiatric condition (*n* = 847 max)	Mean (SD) for patients reporting a psychosomatic or psychiatric misdiagnosis (*n* = 376 max)	*P* value of Kruskal–Wallis test
Satisfaction with medical support	57.50 (29.58)	45.10 (27.65)	**<0.001**
Trust in own clinicians	64.58 (30.45)	54.06 (30.60)	**<0.001**
Trust in clinicians in general	60.75 (26.85)	47.85 (27.44)	**<0.001**

Scales were from 0–100 with 0 signifying no trust/no satisfaction to 100 signifying complete trust/satisfaction.

Both clinicians and patients identified that diagnostic difficulties could impact future medical relationships with patients feeling hurt or ‘*terrified*’ (Ppt 908, SLE, Australia), and acknowledged that patients may sometimes therefore appear defensive and confrontational: ‘*They can be more difficult to manage*’ (Ppt 1, rheumatologist, England). Other patients reported being overly submissive following their adverse experiences: ‘*had the feistiness knocked out of me*’ (Ppt 47, Vasculitis, Wales). There were also indications that medical relationships could become more transactional as opposed to therapeutic, with previously misdiagnosed patients not expressing their feelings due to increased fear of the power differential:

‘You can’t p*ss them off … because they are the only ones who can write that script’ (ppt 103, RA, England)

**Figure 3.**
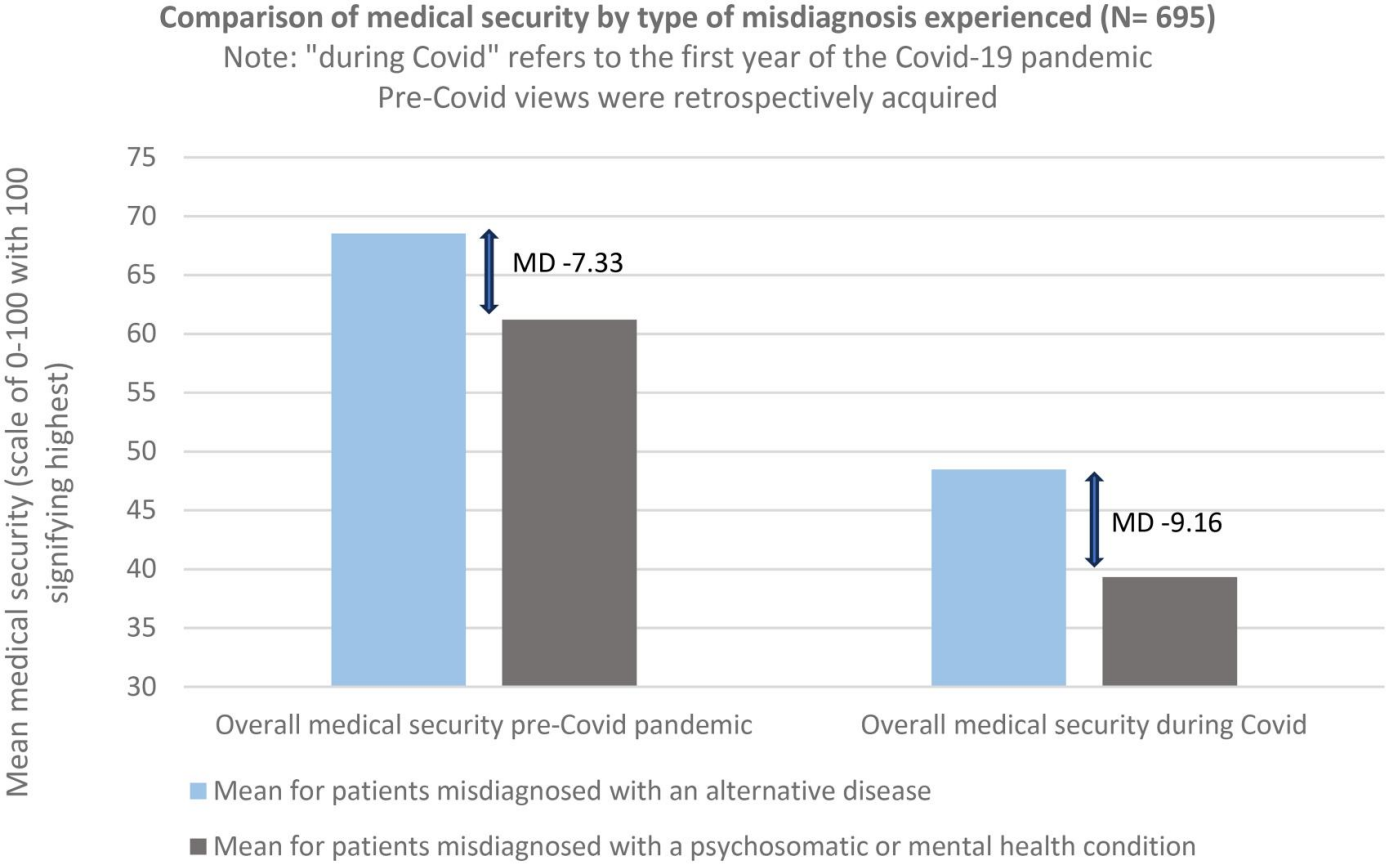
Medical security including the influence of the Covid-19 pandemic (LISTEN cohort)

Medical security was significantly lower in those reporting psychosomatic/psychiatric misdiagnoses as shown in Fig. 3 (*P* < 0.001 at both time points). Medical security during the early stages of the Covid-19 pandemic was reduced overall from pre-pandemic. Additional exploratory analysis revealed that there was an increase in mean difference from −7.33 (95% CI, −10.90 to −3.75) pre-pandemic to −9.16 (95% CI, −13.32 to −5.00) during the pandemic between those with somatic and psychosomatic/psychiatric misdiagnosis. Suggestions for this increased disparity in medical security included reduced resilience to further difficulties in care in patients with these misdiagnoses.

### Impact on healthcare behaviours

Variable impact of misdiagnoses was found on patient behaviours in the LISTEN quantitative data (Table 4). Although significantly (*P* = 0.012) more patients reporting a previous psychosomatic and/or psychiatric misdiagnosis than those reporting an alternative somatic misdiagnosis were healthcare avoidant (managed major disease themselves), they were also more likely (*P* = 0.009) to report mental health symptoms to clinicians. There was no association found with adherence behaviours. In the INSPIRE cohort, patients reporting psychosomatic and/or psychiatric misdiagnoses were significantly more likely to report underplaying both physical and mental health symptoms (both *P* < 0.001) than the rest of the patient study population (Table 5).

**Table 4.** Comparison of healthcare behaviours between different types of misdiagnoses (LISTEN)

Category: healthcare behaviours	Mean for patients misdiagnosed with an alternative somatic disease (*n* = 348) (SD)	Mean for patients misdiagnosed with a psychosomatic and/or psychiatric health condition (*n* = 347) (SD)	Mean difference (SE)	95% confidence interval	*P* value of *t*-test
Report all symptoms	2.59 (1.10)	2.49 (1.21)	−0.10 (0.09)	−0.28, 0.07	0.241
Report MH symptoms	1.33 (1.25)	1.63 (1.18)	0.30 (0.11)	0.07, 0.52	**0.009**
Manage major symptoms without seeking support	2.15 (0.99)	2.35 (1.00)	0.19 (0.08)	0.04, 0.34	**0.012**
Adhere to medication advice	3.76 (0.53)	3.70 (0.57)	−0.06 (0.04)	−1.40, 0.03	0.200
Report any non-adherence to clinician	2.81 (1.46)	2.70 (1.45)	−0.11 (0.16)	−0.41, 0.20	0.502

Frequency scales were from 0 (never) to 4 (always).

**Table 5.** Comparison of over/under playing symptoms between those reporting and those not reporting a psychosomatic or psychiatric misdiagnosis (INSPIRE)

Symptom reporting behaviour frequencies	Mean (SD) for patients not misdiagnosed with a psychosomatic/MH condition	Mean (SD) for patients reporting feeling misdiagnosed with a psychosomatic or mental health condition	*P* value of Kruskal–Wallis test
Underplaying MH symptoms	2.15 (1.91)	2.74 (1.97)	**<0.001**
Underplaying physical health symptoms	1.66 (1.66)	2.12 (1.70)	**<0.001**
Overplaying MH symptoms	0.22 (0.79)	0.23 (0.84)	0.8596
Overplaying physical health symptoms	0.29 (0.85)	0.32 (0.94)	0.6770

Frequency scales were from 0 (never) to 6 (always).

These quantitative findings of greater symptom under-playing by those with previous psychosomatic misdiagnoses was discussed in many interviews:

‘*The long-term effects have been stark. I am too slow to seek medical help* [including for a stroke] *because I don’t want to be seen as a hypochondriac … I am trying to re-educate myself out of this. My current GP surgery have never said this and seem supportive, but from my 20s up until about 36 years old is a long stretch of being told you are making things up or imaging things. That’s a 15 year cycle to break and sometimes I don’t quite manage it*’ (Ppt 91, SLE, Wales)

The impact of these misdiagnoses, with trust as a likely mediator, was felt by some clinicians to adversely affect healthcare behaviours, including symptom reporting, adherence to medication and to advice:

‘*They lose trust in anything that anyone says … you are trying to convince them that something is OK, and they will say yes but a doctor before said that and was wrong*’ (Ppt 7, Gp, England)

Whilst patients reporting psychosomatic and/or psychiatric misdiagnoses had lower trust and satisfaction, there was no association found between reporting behaviours and trust (INSPIRE cohort). Some patients, however, did report during interviews that loss of trust had resulted in self-medication, and/or reduced medication adherence:

‘*“It’s psychosomatic, it’s in your mind” has damaged my trust and courage in telling doctors very much. I even stopped taking my immunosuppressive medicine because of those words*’ (Ppt 123, Sjögrens, England)

Patients overall reported very rarely over-playing their symptoms. However, clinician interviewees suggested that some patients with traumatic diagnostic journeys may find it more difficult to accurately report symptoms:

‘*They* [the previously misdiagnosed] *are the more anxious patients, they can take a while to learn to share their symptoms appropriately. You get people who over-share, who think absolutely everything is related to their lupus and that’s absolutely understandable when they’ve been told nothing is wrong … they don’t trust themselves and run everything past you. And then you have those who took so long to get a diagnosis that you’re constantly coaxing stuff out of them because they’ve been told for so long that nothing is relevant, so they now keep it to themselves*’ (Ppt 140, nurse, England)

### Impact on mental wellbeing

In the LISTEN cohort, patients reporting psychosomatic and/or psychiatric misdiagnoses had significantly lower ratings for every measure of mental wellbeing. WEMWBS scores were 43.49 in those misdiagnosed with an alternative somatic disease compared with 40.01 those with a psychosomatic/MH misdiagnosis (MD −3.49, 95% CI −4.92, −2.05, *P* < 0.001). There was no correlation between length of diagnostic journey and WEMWBS (r = −0.02, *P* = 0.38) and no difference in WEMWBS between those diagnosed <1 year and those whose diagnosis took >10 years from first symptom onset.

In the INSPIRE cohort, participants reporting a psychosomatic and/or psychiatric misdiagnosis (*n* = 376) had significantly higher levels of depression, with a mean PROMIS score of 19.80 (SD = 0.43) compared with 17.03 (SD = 0.23) from other participants (Z = −5.801, *P* < 0.001). Similarly with anxiety, where those reporting a psychosomatic/psychiatric misdiagnosis had significantly higher levels of anxiety (Ζ = −4.984, *P* < 0.001), with a mean GAD 7 score of 7.51 (SD = 0.27) compared with 6.18 (SD = 1.15).

Longer-term repercussions on mental health were also highly apparent. This was evident in the patient reports, their distress in discussing these misdiagnoses, and the finding that 72% reported that these diagnoses still upset them (Fig. 1). This included patients who reported having a misdiagnosis many years ago.

### Thematic analysis of qualitative data

Four themes were generated from the qualitative data: (1) self-blame, self-doubt and reduced self-esteem; (2) psychosomatic/psychiatric misdiagnoses *causing* psychosomatic/psychiatric symptoms; (3) clinician challenges in identifying the ‘[autoimmune] *needle in the* [psychosomatic/psychiatric] *haystack*’; and (4) ameliorating and avoiding the impact of psychosomatic/psychiatric misdiagnoses.

### Theme 1: Self-blame, self-doubt and reduced self-esteem

Over 80% of patients reporting a psychosomatic or psychiatric misdiagnosis stated that it had damaged their self-worth (Fig. 1A, *n* = 347), and the severity and persistence of this damage was frequently raised in interviews:

‘*It has affected my mental health very negatively and I do think it’s affected me in my like sense of self … it’s not good for anyone at any age but as a teenage girl being told you don’t know your own feelings is absolutely no way to shape a human being … I protect myself all the time … and the fury that I feel all the time*’ (Ppt 1159, SLE, Ireland)

Most patients talked about how difficult it was to recover psychologically from the multitude of negative feelings about themselves engendered by a psychosomatic/psychiatric misdiagnosis. This was particularly apparent in those whose early medical interactions had induced feelings of guilt or a view that they were to blame for their symptoms.

‘*I don’t deserve help because this is a disease I’ve brought on myself. You go back to those initial diagnosis, you’ve always got their voices in your head, saying you’re doing this to yourself. You just can’t ever shake that. I’ve tried so hard … it’s always the negatives that you look back on. I just hold onto it. If so many doctors have said it’s my fault, then one person who says it’s not your fault and you have no control over it, it’s a really small voice when you’ve had much larger voices saying, it’s all your fault, you’ve brought this on yourself*’ (Ppt 701, Sjögrens, England)

### Theme 2: Psychosomatic/psychiatric misdiagnoses *causing* psychosomatic/psychiatric symptoms

Receiving a psychosomatic/psychiatric (mis)diagnosis could in itself create/exacerbate psychiatric and psychological symptoms:

‘*One doctor told me I was making myself feel pain and I still can’t forget those words. Telling me I’m doing it to myself has made me very anxious and depressed*’ (Ppt 724, multiple SARDs, England)

There were many examples given of perceived misdiagnoses causing depression and anxiety. One patient reported that a friend had committed suicide with the psychosomatic misdiagnosis (many years before but causing persisting and unresolved psychological damage) given as a reason in her suicide letter, and several others reported having active suicide ideation or suicide attempts:

‘*The [past] disbelief of medics really added to my poor medical health and when a rheumatologist dismissed me I was already suicidal, this just threw me over the edge. Thankfully I am terrible at killing myself, it’s so much more challenging than you think. But the dreadful dismissiveness of doctors when you have a bizarre collection of symptoms is traumatizing and you start to believe them that it’s all in your head*’ (Ppt 108, SLE, England).

The word ‘prove’ was frequently used by patients and clinicians: ‘*That concept that they’ve had to prove they are unwell …* [results in] *tremendous mistrust and anxiety*’ (Ppt 10, rheumatologist, England). The burden of ‘proof’ for often invisible symptoms usually fell to the patient. Combined with the common perception that clinicians had missed or misdiagnosed earlier symptoms, this could lead to patients feeling that they were the only one responsible for closely monitoring their own symptoms. This was felt to generate hypervigilance in some patients, and ironically cause the very psychosomatic symptoms that they had initially been misdiagnosed with:

‘*It’s almost like they* [the previously misdiagnosed] *have got something to prove, they’re angry and anxious … it makes them focus more on symptoms subconsciously*’ (Ppt 17, rheumatologist, Wales)

However, this was also often considered to be a trauma response to initial diagnostic difficulties, and some clinicians postulated that these presentations might also be directly attributable to the disease process, and/or as a result of coping with initially unexplained symptoms:

‘*People are told it’s because they are anxious that they’re having these symptoms but actually I think it’s because they’re having the symptoms they’re experiencing the anxiety*’ (Ppt 52, psychiatrist, England)

### Theme 3: Clinician challenges in identifying the ‘[autoimmune] *needle in the* [psychosomatic/psychiatric] *haystack*’ (ppt 5, GP, England)

Several clinicians stated that earlier referrals for more patients for the most common presenting complaints of joint pains, rashes and fatigue would not be appropriate or financially feasible due to the relative rarity of rheumatic diseases:

‘*For every 100 patients with these types of initial vague symptoms a very small number actually have an underlying pathology*’ (Ppt 21, GP, England)

GP participants also discussed how quicker and more thorough initial investigations would unnecessarily distress the patients with a genuine psychosomatic and/or psychiatric condition, which were reported by clinicians as being much more common than rheumatic diseases.

When the clinician participants were informed of the common feeling of symptoms being disbelieved and patients using ‘in your head’ and ‘disbelieved’ type terminology, they felt it was likely misunderstandings, miscommunication and lack of available treatments rather than clinicians’ intent:

‘*There’s a big difference between not believing the symptoms and believing the symptoms but there not being a cure for them*’ (Ppt 4, rheumatologist, England)

### Theme 4: Ameliorating and avoiding the impact of psychosomatic/psychiatric misdiagnoses

Very few patients (16% of the LISTEN cohort) had been offered support from their clinicians in overcoming the psychological damage from a misdiagnosis, and many were tangibly upset and/or expressed their anger about this in this study. This included patients who reported being misdiagnosed many years previously, were now correctly diagnosed and receiving appropriate care yet had not been given the opportunity to voice their distress:

‘*I still get stressed and weepy … My GP told me I was depressed when in fact I was having seizures … This has caused me so much stress and upset and anger. I’m still very angry and am crying now as I type. I have nowhere to voice this anger*’ (Ppt 574, IA, England)

For some patients, discussing the emotions engendered was a healing process, particularly if the discussion was with the clinician concerned and they responded with empathy:

‘*On one occasion, having been badly gaslit by a clinician I had a further telephone conversation with her and I actually told her that I felt gaslighted … She was shocked and had no idea … She was great. Took it on the chin. Listened and heard. Apologised profusely … For me, the scar of the original encounter was transformed into something much more positive. Hopefully our chat will translate into a more insightful way of dealing with patients*’ (Ppt 820, SLE, England)

As this participant suggested, candid conversations could also lead to more clinician awareness of the impact on all patients. Many rheumatologist interviewees had not considered the ongoing impact: ‘*it’s not something I’ve really thought about*’ (Ppt 16, rheumatologist, England), or they acknowledged the impact but either felt under-qualified to discuss it and/or that it wasn’t a priority in time-constrained clinics. Nurses and psychiatrists were particularly aware and understanding of the extent of the psychological damage, and the need to support and re-build trust in the previously misdiagnosed:

‘*With a new patient we’ll talk about it and how they got diagnosed and just give them that time and space to talk about it … Something I choose to do but it’s not formally done*’ (Ppt 14, nurse, England)

Patients described how the trauma from previous misdiagnosis was reduced by certain clinician behaviours, including explicitly demonstrating belief in their symptoms, being readily available: ‘*Very rare that I don’t get a call back within the day … he’s never let me down*’ (Ppt 448, SLE, England), and being aware of the trauma and advocating for the patient. In addition, some patients described how they had changed their own behaviour and reactions, and decided not to ‘take it personally’. This resilience was discussed by some as becoming easier with time and knowledge, but others reported that it became more challenging due to the trauma of repeated misdiagnoses, and several described PTSD-type reactions.

The importance of correcting misattributions on medical records once the correct diagnosis was reached was highlighted. This was reported to rarely occur, leaving patients feeling vulnerable to—and many reported receiving—repeated misdiagnoses.

‘*Once I said I was anxious because all of the odd things, new symptoms,* [that] *were happening to me next thing I know I am diagnosed with a general anxiety disorder. Then once docs saw that … all my symptoms were due to my anxiety*’ (Ppt 865, multiple SARDs, US)

## Discussion

This study presents substantial quantitative and qualitative evidence that psychosomatic and psychiatric misdiagnoses are strongly associated with persisting adverse influences on multiple areas of patients’ lives, including satisfaction with medical care, mental health and self-worth. Our study also provides evidence that certain types of misdiagnoses, particularly those perceived to represent clinician disbelief, show stronger associations with future mental wellbeing than the length of diagnostic delay. Although patients reporting long diagnostic delays due to misdiagnoses with alternative somatic diseases may accrue significant physical damage [6, 12], psychosomatic or psychiatric (mis)diagnoses appear to often cause additional deep and enduring damage to patients’ sense of self and worth. Multiple studies on other populations have reported healthcare avoidance due to previous negative medical experiences [30, 31]. Our study also found increased healthcare avoidance and a higher propensity to under-report symptoms in patients reporting psychosomatic and/or psychiatric misdiagnoses, although our quantitative data demonstrated no association with medication adherence. In common with our previous study, we identified that difficult diagnostic journeys had an adverse impact on self-worth [21]. This extended in some cases to self-blame for the symptoms, and to suicidal thoughts and suicide attempts in some patients. We suggest that the frequent (mis)diagnosis of health anxiety for patients presenting with a multitude of initially unexplained symptoms may be better explained as an often-persisting health *system* anxiety. This is where some patients remain in a perpetual state of—understandable and experience-based—anxiety that current health systems will not have the knowledge base or resources to correctly diagnose and treat their symptoms. Our study suggests that this health system anxiety may in turn *cause* symptoms and healthcare behaviours that can perpetuate and seemingly validate the original misdiagnoses, yet are partially or wholly iatrogenic.

We considered the relevance of the Patient Health Engagement (PHE) model [19] and attachment theory [17, 18] for SARD patients to explore whether these existing theories could assist in explaining our findings in relation to the impact of misdiagnoses. Our study indicates that perceived psychosomatic and/or psychiatric misdiagnoses can prevent patients reaching the final ‘Eudaimonic’ [20] phase of the PHE model, as these misdiagnoses reduce confidence in themselves and clinicians, and patients’ ability/willingness to ‘activate healthcare professionals when needed’ [20]. Many patients therefore remained in (or returned to through subsequent misdiagnoses) the earlier phases of ‘blackout’ and ‘arousal’ in the PHE model [20], with adverse impacts on mental health and healthcare behaviours. Although attachment theory more usually refers to the parent–child relationship, clinicians can become the ‘secure base’ in times of medical vulnerability. We found that the core components of a secure attachment as summarised by Duschinsky *et al.* [17] were frequently challenged by psychosomatic and psychiatric misdiagnoses. This included reduced belief in continued worthiness of care, and reduced confidence in the attachment figure’s good intentions and accessibility. Bennett and colleagues also investigated attachment in lupus patients and found that attachment avoidance was negatively associated with medication adherence, and that attachment anxiety was negatively related to QoL [32]. Also of relevance is Klest’s report on how patients who have experienced trauma ‘high in betrayal (where the perpetrator and the victim have a close relationship)’ have higher levels of depression and future difficulties in forming trusting relationships, including with clinicians [33]. This was particularly apparent in our participants whose ‘betrayal trauma’ was felt to be directly from their medical interactions.

In agreement with another study [34], we identified that loss of trust extended beyond the clinician who made the perceived misdiagnosis. Our findings are very much in agreement with Rustad *et al.* that ‘acknowledgment that trust has been fractured’ is essential before re-building trust [35], and supports Suzuki’s calls for trust-enhancing interventions specifically for the misdiagnosed [34]. However, most clinicians and health services lacked the resources to offer support in overcoming these adverse medical experiences. Skirbekk *et al.*’s discussion of how the implicit ‘mandate of trust’ between patients and clinicians may need to be more explicit in complex diseases [36] is highly relevant to SARDs, particularly in the previously misdiagnosed. However, given the variable clinician knowledge of SARDs, inadequacy of SARD diagnostic tests [23] and the high quantity of misdiagnoses, a degree of mistrust is not always a negative as it can act as a facilitator to patient involvement [37], and protect the patient from poor medical decisions.

Graber *et al.* describe three categories of diagnostic errors in medicine: ‘no-fault errors’ which include atypical presentations or diseases initially presenting as something more common [38], ‘system errors’ where these errors are driven by issues in the healthcare system such as limited resources to run tests and ‘cognitive errors’ which are to do with ‘faulty data collection/interpretation, flawed reasoning, or limited knowledge’ [38]. There will always be a margin of error when it comes to making clinical diagnoses, especially in systemic autoimmune disease where all these types of errors are prevalent, and systemic autoimmunity was reported as being ‘the autoimmune needle in the psychosomatic and psychiatric haystack’. It is also important to acknowledge that what the patient perceives the clinician to believe (or often more importantly, to disbelieve) during a clinical interaction may be at odds with the clinician’s intent. Clinicians were often unaware that offering ‘reassurance’, although well-intentioned, may be perceived as dismissive. This highlights the importance of improved clinician–patient communication, and better management of uncertainty and initially unexplained symptoms.

A ‘restorative action and reconciliation’ process was proposed by our patient collaborators, as part of wider training on effective communication with patients. This would incorporate those patients who perceived that they have received a misdiagnosis being given the opportunity to discuss honestly the emotions engendered, and receive explicit acknowledgement of the trauma experienced, ideally from the (mis)diagnosing clinician. For those where the clinician–patient partnership is continuing, this would form part of the ongoing rebuilding of trust and understanding process. However, the current UK and other countries healthcare institution procedures and fears of repercussions [39] may discourage openness in clinicians (and patients) reporting or discussing errors, or in challenging potential mistakes and misdiagnoses made by their colleagues.

Psychosomatic and psychiatric misdiagnoses not only caused distress and delays in diagnosis, but their permanence on medical records, even once the correct diagnosis had been achieved, left many patients in fear of future disbelief and vulnerable to repeated confirmation bias. Previous research supports our participant fears and experiences [5], including detailing that clinicians presented with a potential diagnosis are significantly less likely to reach the correct diagnosis [40]. A ‘disconfirmatory’ or balanced search for alternative evidence (including listening to the patients’ views), could lead to a higher rate of correct diagnoses [41]. One contributing factor to frequent misdiagnosis in SARDs may be the belief held by some physicians, as reported in the literature, that: ‘*A long list of symptoms should therefore be a “red flag” that the presenting symptom will not be “explained by disease”*’ [42]. It is essential to consider the caveat that a long list of symptoms can also be a red flag indicating systemic autoimmunity. Bransfield instead proposes that in complex multisystem diseases, ‘unexplained’ symptoms may often just be unexamined and/or not within the assessing clinician’s sphere of knowledge. To reduce the frequent misdiagnoses observed in many conditions, including SARDs, he states that ‘*If a complex illness with a multitude of both mental and physical components begins later in life, the likelihood that this is an immune mediated, multisystem disorder is greater than it being a psychosomatic disorder*’ [43]. It is also important to recognize that many patients with chronic diseases have a high degree of ‘attributional insight’ into their own symptoms [44]. Neither patient nor clinician will always be correct with regard to attribution, but eliciting and valuing patient views as to whether a symptom is more likely to be psychosomatic or somatic can assist in correct diagnoses [44] and in building mutual trust.

This research contributes to the debate regarding the attribution of neuropsychiatric symptoms, such as depression and anxiety, in SARDs [23], by suggesting that some symptoms may be caused or exacerbated by misdiagnosis of an underlying SARD and thus be partially or wholly iatrogenic. Attributional difficulties are further compounded by the limitations of current health systems for diagnosing and managing patients with systemic autoimmunity whose symptoms frequently cross speciality borders. Such a situation makes clear the importance of careful management of patients presenting with a possible SARD with co-occurring psychological and/or psychiatric symptoms. It implies at least three distinct steps on the part of the clinician are necessary. First, a recognition that neuropsychiatric symptoms are a common and direct manifestation of SARDs [22] and other autoimmune diseases, including as a prodrome to full disease/flare onset [45, 46]. Second, an assessment by appropriately qualified specialists of the multiple potential indirect causes of psychological and/or psychiatric symptoms in patients with chronic diseases including poor quality of life, the effects of medications and adverse medical experiences [28]. Third, an appreciation that psychosomatic and/or psychiatric misdiagnoses are common in SARDs. This includes the necessity of acknowledging, and assisting in ameliorating the possible persisting impact of perceived misdiagnoses on patients, including on medical relationships and the under-reporting of symptoms. As Clark insightfully writes from personal experience: ‘rebuilding trust with patients who have been previously misdiagnosed requires time, compassion, and empathy’ [47]. Improved mutual understanding of the other party in the medical relationship’s thought processes is essential as it was clear from the interviews that there is currently a chasm of misunderstanding and miscommunication between clinicians and SARDs patients.

## Strengths and limitations

We cannot make any inferences as to the direction of cause and effect from our data. For example, we do not know whether participants who reported a psychosomatic and/or psychiatric misdiagnosis were statistically significantly more likely to become depressed and/or anxious *because* of the misdiagnosis, or if they had higher rates of pre-morbid anxiety and depression which may have contributed to their perceived misdiagnosis. We had no means of confirming participant reports of past misdiagnoses, and were therefore reliant on their reports and views as to the nature of the perceived misdiagnosis. As several clinician participants highlighted, some patients may have misinterpreted initial diagnostic uncertainty as insinuating a psychosomatic cause when this was not the clinician’s intention, although this also suggests the need for improved communication of diagnostic uncertainty. Self-reports are also subject to multiple potential biases including recall, confirmation and anchoring bias. There were multiple potential confounders, and the low numbers of certain groups of respondents (e.g. males) reduces the generalizability of the results. Although males are in the minority in some SARDs such as SLE and Sjögren’s, our proportions were not fully representative of the wider SARD population. This lower proportion of hard-to-reach groups is common in research [48], and also a reflection of our online recruitment strategy. We have now employed an inequalities researcher to ensure our future studies are more representative and that we receive more views and priorities from under-served groups. Excluding participants who marked ‘unsure’ as to whether they perceived they had a previous misdiagnosis may have skewed the results towards those who had been most affected by a misdiagnosis.

Study strengths included using the data from two large-scale SARD studies, and including both patient and clinician interviews to assist in further exploring and explaining the quantitative data acquired. Concordance between the two types of data and between the two studies reduced threats to validity, and investigation of any discordance led to deeper, more nuanced understanding, thus highlighting the value of mixed methods research [49]. For example, there was a discrepancy between the qualitative data suggesting changes in medication adherence from adverse medical experiences, and the quantitative data showing limited/no impact. In agreement with previous research, an explanation may be that previously misdiagnosed patients’ behaviours may be more skewed to both extremes, thus balancing each other out in the statistical findings [28]. An additional strength of this study team is the inclusion of patients as equal and valued members of the study team, with input at every stage of the research cycle including as co-authors.

## Conclusion

Patients who reported having received previous psychosomatic and/or psychiatric misdiagnoses have higher adverse outcomes in multiple domains of wellbeing, medical relationships and some healthcare behaviours. Our evidence suggests that clinicians should explicitly acknowledge previous misdiagnoses, discuss and empathize with their patients as to the potential ongoing impacts and offer targeted support to reduce the persisting negative impacts. Health services ensuring greater access to psychologists and talking therapy for patients reporting previous psychosomatic or psychiatric misdiagnoses could reduce the identified long-term adverse repercussions to health, healthcare behaviours and medical relationships. Misdiagnoses may be reduced by education encouraging clinicians to consider systemic autoimmunity when patients present with multiple, seemingly unconnected, physical and mental health symptoms.

## Data Availability

Anonymized data will be available on reasonable request following the completion of the INSPIRE studies.
